# A Cross-Species Study of PI3K Protein-Protein Interactions Reveals the Direct Interaction of P85 and SHP2

**DOI:** 10.1038/srep20471

**Published:** 2016-02-03

**Authors:** Susanne B. Breitkopf, Xuemei Yang, Michael J. Begley, Meghana Kulkarni, Yu-Hsin Chiu, Alexa B. Turke, Jessica Lauriol, Min Yuan, Jie Qi, Jeffrey A. Engelman, Pengyu Hong, Maria I. Kontaridis, Lewis C. Cantley, Norbert Perrimon, John M. Asara

**Affiliations:** 1 Beth Israel Deaconess Medical Center, Division of Signal Transduction, Boston, MA USA; 2 Harvard Medical School, Department of Medicine, Boston, MA USA; 3 Centers for Therapeutic Innovation, Pfizer, Boston, Massachusetts, USA; 4 Howard Hughes Medical Institute, Harvard Medical School, Department of Genetics, Boston, MA USA; 5 Novartis, Cambridge, MA USA; 6 Massachusetts General Hospital, Center for Thoracic Cancers, Charlestown, MA USA; 7 Beth Israel Deaconess Medical Center, Division of Cardiology, Boston, MA USA; 8 Brandeis University, Department of Computer Science, Waltham, MA USA; 9 Department of Biological Chemistry and Molecular Pharmacology, Harvard Medical School, Boston, MA USA; 10 Cancer Center, Weill Cornell Medical College, New York, NY USA

## Abstract

Using a series of immunoprecipitation (IP) – tandem mass spectrometry (LC-MS/MS) experiments and reciprocal BLAST, we conducted a fly-human cross-species comparison of the phosphoinositide-3-kinase (PI3K) interactome in a drosophila S2R+ cell line and several NSCLC and human multiple myeloma cell lines to identify conserved interacting proteins to PI3K, a critical signaling regulator of the AKT pathway. Using H929 human cancer cells and drosophila S2R+ cells, our data revealed an unexpected *direct* binding of Corkscrew, the drosophila ortholog of the non-receptor protein tyrosine phosphatase type II (SHP2) to the Pi3k21B (p60) regulatory subunit of PI3K (p50/p85 human ortholog) but no association with Pi3k92e, the human ortholog of the p110 catalytic subunit. The p85-SHP2 association was validated in human cell lines, and formed a ternary regulatory complex with GRB2-associated-binding protein 2 (GAB2). Validation experiments with knockdown of GAB2 and Far-Western blots proved the direct interaction of SHP2 with p85, independent of adaptor proteins and transfected FLAG-p85 provided evidence that SHP2 binding on p85 occurred on the SH2 domains. A disruption of the SHP2-p85 complex took place after insulin/IGF1 stimulation or imatinib treatment, suggesting that the direct SHP2-p85 interaction was both independent of AKT activation and positively regulates the ERK signaling pathway.

Proteomics analyses across species can help reveal biologically conserved pathways and protein interactions. Cross-species comparisons have been performed from protein-protein interaction (PPI) networks using different bioinformatics based approaches combining conserved network substructures with shared interaction domains^1^,^2^,^3^,^4^,^5^,^6^ and phosphorylation network datasets^7^ using sequence alignment. The cross-species approach can be useful for interrogating biologically important complexes within PPI datasets since mass spectrometry approaches often produce an excess of non-specific proteins that are not part of the core complex for biological activity^8^.

Here, we used a comparative PPI strategy to identify proteins surrounding the p85 regulatory subunit of phosphoinositide-3-kinase (PI3K), a critical lipid kinase in cellular signaling events leading to cell proliferation, and growth^9^. The class I PI3K protein kinase is made up of two subunits, a catalytic subunit (p110α, p110β or p110δ) and a regulatory subunit (p85α, p85β and p55γ)^10^, and its main function is to phosphorylate phosphoinositol-4,5-bisphosphate (PIP2) to produce phosphoinositol-3,4,5-trisphosphate (PIP3). Growth factor stimulation of receptor tyrosine kinases (RTKs) leads to the recruitment of adaptor proteins such as the GRB2-associated binding proteins GAB1 and GAB2, the insulin receptor substrates IRS1 and IRS2, and p85 itself to the membrane. The Src homology 2 (SH2) domains on p85 can bind to the phosphotyr osine residue motif “pYXXM “and the SH3 domains on p85 can bind to RTKs or adaptor proteins^11^,^12^. The heterodimer of p85 and p110 has a very strong affinity via coiled-coil interactions between the inter-SH2 domain of p85 and the amino terminal adaptor binding domain (ABD) of p110. When in excess over p110 proteins, p85 proteins exist as homodimers that can interact with other proteins, including PTEN and BRD7^13^,^14^,^15^. These ‘free p85’ proteins can compete with p85/p110 heterodimers for binding to upstream activators^16^,^17^ and thereby suppress activation of PI3K. The product of PI3K, PIP3, acts as a cellular second messenger and binds to the pleckstrin homology (PH) domain of the serine/threonine kinase AKT as well as to other signaling proteins, triggering many cellular events including mTOR activation which leads to cell proliferation and tumor growth^18^,^19^. Mutations of PIK3CA, the gene encoding p110α, can result in sustained activation of PI3K signaling and are often involved in diseases such as cancer^9^,^20^,^21^,^22^.

The protein tyrosine phosphatase SHP2 (encoded by PTPN11) plays a complex role in growth factor signaling. The SH2 domains can bind to Tyr-phosphorylated adaptor proteins such as GAB1 and GAB2 and, in the presence of a phosphorylated YXXN motif, SHP2 can bind to GRB2 to initiate activation of the Ras-MAPK signaling pathway^21^,^22^,^23^. In addition, SHP2 can dephosphorylate certain growth factor receptors and adaptors such as GABs to either enhance or limit growth factor signaling. Further, it is known that germline mutations in *PTPN11* can result in LEOPARD and Noonan syndromes, characterized by a variety of disorders^23^,^24^. While the role of SHP2 in the PI3K pathway is not well-defined, it has been reported that SHP2 co-immunoprecipitates with p85^25^,^26^,^27^,^28^ and SHP2 can regulate the level of AKT signaling in an epidermal growth factor (EGF) dependent manner^26^. While a complex with p85 and SHP2 is not novel, its function and binding mode is not well understood.

Here, we show with both mass spectral and biochemical evidence that SHP2 *directly* and preferentially binds to the p85 regulatory subunit of PI3K in both *drosophila* and human cell lines and evaluate its function. We identified the p85-SHP2 interaction through PI3K complex IPs from overlapping immunoprecipitations (IP) and microcapillary liquid chromatography/tandem mass spectrometry (LC-MS/MS) experiments, from *drosophila* S2R+ cells and a variety of human lung cancer and multiple myeloma cell lines using reciprocal BLAST searches^29^,^30^,^31^. Specifically, we overlaid the bait-prey interaction data from IP-MS of the Pi3k21B (p60) regulatory subunit and the Pi3k92e (p110) catalytic subunit of PI3K in *drosophila* S2R+ cells with IPs of the p85α regulatory subunit of PI3K human cancer cell lines. While it is known that SHP2 binds directly to several PI3K interacting proteins, the direct interaction of SHP2 with p85 has not been established. Our data show that SHP2 binds directly to free p85 (not the p85/p110 PI3K heterodimer) and that a ternary complex of GAB2-SHP2-p85 impairs PI3K signaling while enhancing MAPK signaling.

## Materials and Methods

### Human Cell lines

Non-small cell lung cancer (NSCL) lines EBC-1, HCC827, A549, H1993 and H1703 were obtained from the Jeffrey Engelman laboratory, Mass General Hospital (MGH)) and maintained in Dulbecco’s Modified Eagle’s medium (DMEM, Sigma-Aldrich) supplemented with 10% FCS, 100 units/mL penicillin and 100 units/mL streptomycin, the multiple myeloma RPMI-8226, H929 cell line (Kenneth Anderson, Dana-Farber Cancer Institute, ATCC), leukemia cell line K562 (Pier Paolo Pandolfi, Beth Israel Deaconess Medical Center (BIDMC), ATCC) were maintained in RPMI 1640 (Cellgro, Mediatch Inc) supplemented with 10% FCS, 100 units/mL penicillin and 100 units/mL streptomycin, multiple myeloma cell line MOLP8 (DSMZ) were maintained in RPMI 1640 (Cellgro, Mediatch Inc) supplemented with 20% FCS, 100 units/mL penicillin and 100 units/mL streptomycin, 293T cells were maintained in Dulbecco’s modified Eagle’s medium supplemented with 10% FCS, 100 units/mL penicillin and 100 units/mL streptomycin. *Drosophila* S2R+ cells were grown in Schneider’s media.

### Kinase Inhibitor drugs

Imatinib (1 μM) was purchased from Tocris Bioscience. BKM-120 (5 μM) was purchased from Active Biochemical Co. dimethyl sulfoxide (DMSO) was purchased from Sigma-Aldrich. NVP-AEW541 (5 μM) was purchased from Selleck. Bortezomib (50 nM) was purchased from LC Laboratories, GSK11220212 (250 nM) was provided by the Cantley lab (BIDMC). Cells in normal growth conditions, 70% confluence, were treated with inhibitors for one hour prior to lysis using DMSO as vehicle.

PHA-665752 was purchased from Tocris. Gefitinib (1 μmol/L) were obtained from American Custom Chemical. TAE-684 (100 nmol/L) was purchased from Selleck. Rapamycin (50 nmol/L) was purchased from Sigma. Cells in normal growth conditions, 70% confluence, were treated with inhibitors for 6 hour, except for rapamycin, which was used for 16 hours prior to lysis using DMSO as vehicle.

### Growth factor stimulation

Cells were starved under serum free conditions for 16 hours and stimulated by adding insulin (100 μg/mL, Sigma Aldrich), IGF1 (20 ng/mL, Sigma Aldrich), PDGF (20 ng/mL, Austral Biologicals), EGF (200 ng/mL, PeproTech) or FCS (10%, Cellgro, Mediatch Inc) for 90 minutes prior to lysis.

### Drosophila TAP tags and Immunoprecipitation

Stable tandem affinity purification (TAP)-tagged Pi3k21B (regulatory subunit of PI3K, p55/p85 human ortholog) and Pi3k92e (catalytic subunit of PI3K, p110 human ortholog) were created into S2R+ Drosophila cells using the metallothionein promoter^32^ into the C-terminal tag TAP vector and created stable cell lines for each, as well as a control cell line for subtracting nonspecific interactors or contaminants. Cells (1 × 10^9^ to 2 × 10^9^) induced with 140 μM CuSO_4_ overnight were used for each lysis at the given condition. Cells were untreated or treated with insulin for 10 and 30 minutes to stimulate the PI3K pathway. The Drosophila cells were washed with *wash buffer* and lysed as described^33^. In-solution TAP IP and elution was performed as previously described^34^. In short, an antibody against the TAP was used to immunoprecipitate the TAP tagged proteins overnight at 4 °C, the protein A/G sepharose beads (GE Healthcare) were added for the last 2 hours of incubation. Negative control IPs were performed by incubation just A/G sepharose beads and empty TAP vectors with the fly lysate overnight at 4 °C. After incubation, beads were washed 3 times and eluted in ammonium bicarbonate containing buffer. Several micrograms of TAP immunoprecipitation from each bait condition were reduced with 10 mM dithiothreitol at 55 °C, alkylated with 55 mM iodoacetamide at room temperature, and then digested overnight with 2.5 μg of modified trypsin (Promega) at pH 8.3 (50 mM ammonium bicarbonate) in a total of 200 μl. The digestion was stopped with 5% trifluoroacetic acid (TFA) and cleaned of buffer and debris with a C18 ZipTip (Millipore). Thirty-five microliters of aqueous high-performance liquid chromatography (HPLC) A buffer (99% H2O/0.9% Acetonitrile/0.1% Formic acid) was added to the C18 ZipTip elution (50% acetonitrile/0.1% TFA) and was dried to 10 μL in a SpeedVac with no heat to concentrate the sample and remove organic content. Four total IPs were performed from drosophila cells (Pi3k21B basal in S2R+ cells, Pi3k21B insulin in S2R+ cells, Pi3k92e basal in S2R+ cells and Pi3k92e insulin in S2R+ cells). Three biological replicates were performed for each drosophila IP-MS experiment.

### Human Antibody Immunoprecipitation

Human cancer cells were grown in up to five 15 cm^2^ dishes to 80% confluence, washed with PBS and lysed in a 0.5% NP-40 containing lysis buffer (0.5% NP-40, 150 mM NaCl, 10 mM Tris/HCL pH 7.4, 1 mM EDTA pH 8, 1 mM EGTA). Lysates were centrifuged at 16,000 × *g* for 5 minutes at 4 °C. The supernatant was used for subsequent procedures. Co-IPs were done by incubating 1 mg (western blotting)/10 mg (LC-MS/MS) of the cell lysate with 1 μg (western blotting)/10 μg (LC-MS/MS) of antibody against PI3K bait proteins (rabbit polyclonal p85α, Millipore, Cat. # 06-195) in human overnight at 4 °C and 70 μL of protein A/G sepharose beads (GE Healthcare) slurry were added to the lysate antibody solution for the last 2 hours of incubation. Negative control IPs were performed by incubation 70 μL of protein A/G sepharose beads (GE Healthcare) slurry, and 10 μg of rabbit IgG with the 10 mg of H929 lysates overnight at 4 °C. After incubation, beads were washed 3 times with 1 mL of lysis buffer, and boiled in 1X SDS sample buffer containing β-mercaptoethanol. IPs were separated by SDS-PAGE until the 55 kDa pre-stained marker was separated from the band above and below (short gel run, ~1/6 distance of mini gel lane)^11^. After staining with coomassie blue and destaining, gel sections were excised above and below the IgG heavy chain band to avoid antibody contamination and peptide signal suppression. The gel region at 55 kDa generally does not contain useful proteins other than high levels of contaminants such as immunoglobulin and BSA and were excluded from the analysis. Gel sections were reduced with 55 mM DTT, alkylated with 10 mM iodoacetamide (Sigma-Aldrich), and digested overnight with TPCK modified trypsin (Promega) at pH = 8.3. Peptides were extracted, concentrated to 10 μL in a SpeedVac. Sixteen total IPs were performed from human cells (p85 serum H1703, p85 imatinib H1703, p85 serum starved H929, p85 serum H929, p85 serum A549, p85 serum 8226, p85 serum starved 8226, p85 serum bortezomib, p85 serum HCC827, p85 serum gefitinib HCC827, p85 serum gefitinib + HGF, p85 serum H1993, p85 serum PHA665752 H1993). Two biological replicates were performed for each human IP-MS experiment, where possible.

### Tandem Mass spectrometry (LC-MS/MS)

Peptide complexes were analyzed by positive ion mode LC-MS/MS using a hybrid LTQ-Orbitrap XL-ETD mass spectrometer (Thermo Fisher Scientific) via CID with data-dependent analysis (DDA) using a Top 5 approach (1 full FT-MS scan followed by 5 MS/MS CID scans). Maximum injection time was 50 msec for MS and 100 msec for MS/MS with 1 microscan for both modes. Isolation width was 2.3 Da and dynamic exclusion time was set to 90 sec. Peptides were delivered and separated using an EASY-nLC I nanoflow HPLC (Thermo Fisher Scientific) at 300 nL/min using self-packed 15 cm length × 75 μm i.d. C_18_ fritted microcapillary Picofrit columns (New Objective). Solvent gradient conditions were 140 minutes from 3% B buffer to 38% B (B buffer: 100% acetonitrile; A buffer: 0.1% formic acid/99.9% water). MS/MS spectra were analyzed using the Sequest algorithm in Proteomics Browser Software (PBS; Harvard University) and the Mascot 2.3 search engine by searching the reversed and concatenated Swiss-Prot protein database (version 2011_9 containing 53,214 protein sequence entries, http://www.ebi.ac.uk/uniprot/database/download.html) with a parent ion tolerance of 18 ppm and fragment ion tolerance of 0.80 Da. Carbamidomethylation of Cys (+57.0293 Da) was specified in Sequest as a fixed modification and oxidation of Met (+15.9949 Da) and deamidation of Asn/Gln (+0.9840 Da) as variable modifications. Results were imported into Scaffold 4.0 software (Proteome Software) with a peptide threshold of ~85%, protein threshold of 95%, resulting in a peptide false discovery rate (FDR) of ~1.6%. In addition to FDR, a protein was only accepted if at least three spectral counts from at least two unique peptides were present from at least two of the 16 human experiments and at least three spectral counts from one of the four drosophila experiments. Common contaminants including human keratins, dermicidin, milk derived caseins, porcine trypsin and bovine serum albumin were initially removed from the analysis and do not appear in any spectrum count reports. In addition to contaminants, we removed “sticky” prey proteins during the reciprocal BLAST step if they belong to the following categories: ribosomal proteins, heat-shock proteins, actin, and tubulin since we know from years of literature that these proteins are not part of the core PI3K complex.

### PPI Network

Protein reports were exported from Scaffold 3.5 software and imported into Cytoscape v2.6 software (http://www.cytoscape.org/) in order to create a PPI landscape for, p85 and Pi3k21B (fly p85) and Pi3K92e (fly p110) IPs. The spectral counts over each of the analyses were averaged require that the average value for the spectral count was three or better. The directions of the edges point from baits to prey. The edge colors represent the following: black 0 min, green 10 min, and orange 30 min of insulin treatment.

### Reciprocal BLAST

We combined the orthology information downloaded from NCBI HomoloGene (http://www.ncbi.nlm.nih.gov/homologene) and InParanoid (http://inparanoid51.sbc.su.se/cgi-bin/index.cgi) on July 24, 2009. For those genes without orthologs in the above two databases, we used NCBI-Blastp to identify reciprocal best three hits (with E-value < 1e–60 as the cutoff) between human proteomes and *Drosophila* proteomes as the potential ortholog pairs. The protein sequences were downloaded from NCBI on July 24, 2009. We then used the collected orthology information to identify interlogs in *Drosophila* PI3K interaction data and human PI3K interaction data. Common proteins from the reciprocal Blast overlap between at least one human and *drosophila* experiment were retained for further analyses and validation. Proteins that were observed from either drosophila TAP control IPs in SR2+ lysate or the rabbit serum IgG IPs from human H929 lysate were removed from the final results.

### Western blot analysis

Western blot analyses were conducted after separation by SDS-PAGE and transferred to a nitrocellulose membrane. Antibodies against GAB1 (rabbit polyclonal), GAB2 (rabbit polyclonal), GST, FLAG (mouse monoclonal) were purchased from Cell Signaling, Technologies. Antibody against SHP2 (rabbit polyclonal), p110α (rabbit polyclonal) were purchased from Santa Cruz Biotechnology. P85α (rabbit, polyclonal) antibody were purchased from Millipore. All antibodies were used per manufacturer’s instructions. Antibody binding was detected using enhanced chemiluminescence (PerkinElmer).

### FLAG protein constructs

The constructs FLAG-p85 WT, FLAG-p85 w/o iSH2, FLAG-iSH2, FLAG-p85 w/o 1-645 and FLAG-p85 w/o nSH2 was provided by Yu-Hsin Chiu from the Lewis Cantley laboratory (BIDMC). H929 cells with 60% confluence were transfected using Mirus transfection reagent from Mirus Bio LLC following the manufacturer’s protocol for suspension cells. 48 hr after transfection the cells were tested for transfection efficiency by blotting for FLAG by Western Blot.

### Far-Western Blots

For Far-Western blotting experiments, cells were lysed by RIPA buffer (50 mM Tris-HCL, pH 7.4, 1% NP-40, 0.5% sodium deoxycholate, 150 mM NaCl, 1 mM EDTA, 0.1% SDS, 1 μg/ml aprotinin, 1 μg/ml leupeptin, 1 μg/ml pepstatin, 1 mM PMSF, 1 mM Sodium Fluoride, 1 mM Sodium Orthovanadate, 1 mM DTT) followed by a SHP2 or p85 IP. The eluent were run on SDS-Page gel and transferred on nitrocellulose membrane. The membrane was blocked with PBST (4 mM KH2PO4, 16 mM Na2HPO4, 115 mM NaCl (pH7.4), 0,05% Tween-20) containing 3% milk. The membrane was incubated with purified GST-tagged proteins from *E. coli* in PBST. After three brief washes with PBST, the proteins were then blotted with GST antibody followed by secondary antibody. GST-Shp2 wt construct was obtained from the Maria Kontaridis lab at BIDMC. GST-p85 full length, GST-SH2 and GST controls were obtained from the Lewis Cantley lab at BIDMC.

### shRNA Knockdown

293T cells were seeded in 10 cm plates 24 hours prior to experiments with 60–70% confluence. Four shRNA constructs were pooled and transfected into 293T cells with Mirus transfection reagent from Mirus Bio LLC following the provided protocol, as well as empty vector pLKO.1 as a control. The virus was harvested after 36 to 48 hours of transfection. H929 cells with 70–80% confluence were then infected, and selected by 2 μg/ml of puromycin 48 hours post infection. After seven days of constant selection with puromycin, cells were subject for test for knockdown efficiency. shRNA pools for GAB2 (RHS4533-NM_012296) containing four constructs and SHP2 (RHS2533-NM_002834, D13540, L03535, L08807, AU123593) and p85α (RHS3979-9600692, 9600696, 9607293, 9607295) containing four constructs were purchased from Thermo Fisher Scientific.

## Results

### Identification of the p85-SHP2 complex by mass spectrometry

**Figure 1:**
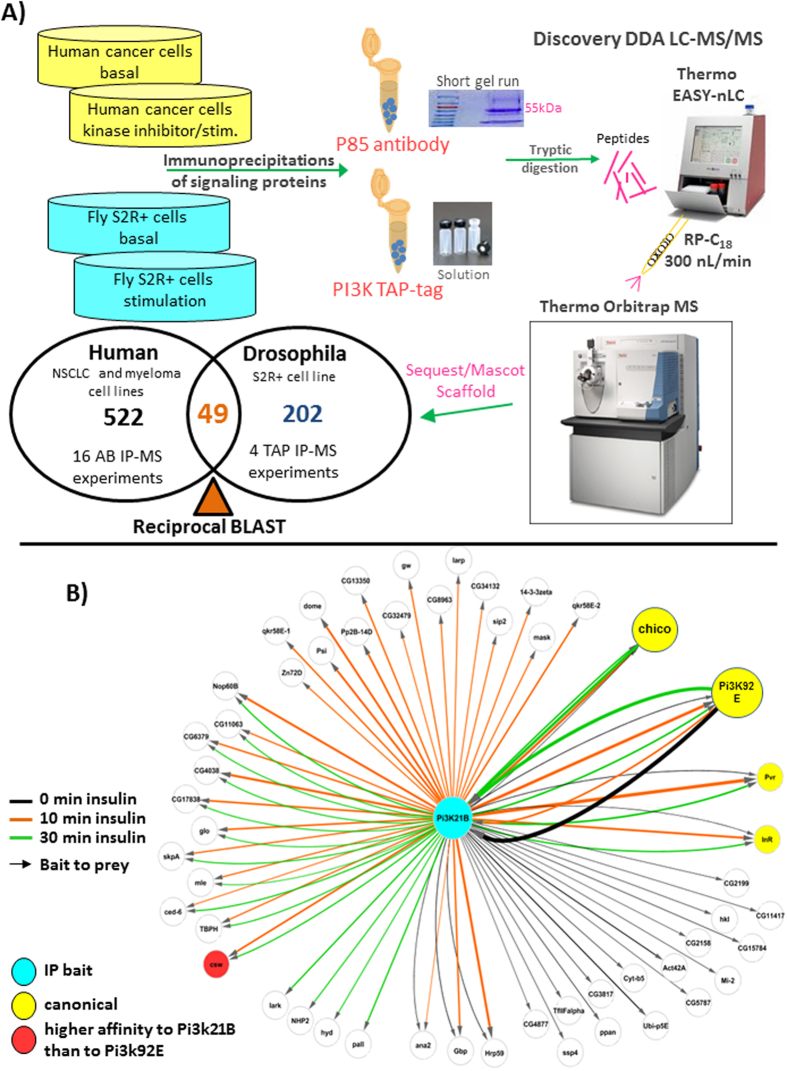
(**A**)The workflow of the cross-species PI3K immunoprecipitation (IP)-LC-MS/MS proteomics strategy from human vs. drosophila cells. The immunoprecipitation, tryptic digestion and high resolution shotgun LC-MS/MS analyses from both drosophila and human cells separately result in the Venn diagram showing the numbers of identified proteins and overlapping proteins. **(B)** The 49 possible PI3K interaction candidates of biological importance under various levels of insulin stimulation from drosophila S2R+ cells.

We have studied the PI3K protein complex over the last several years using LC-MS/MS and protein binding can vary across cancer cell lines due to differences in biological activity, sticky proteins, etc. In order to address the PI3K core complex and its function, *drosophila* S2R+ cells^32^ were used as a model system to identify PI3K interactions that were conserved through evolution from common human cancers back to flies and to help sort out non-specific binding proteins from biological interactions. Four *drosophila* experiments from tandem affinity purification (TAP) tagged Pi3k21B (human p85 ortholog) and Pi3k92e (human p110 ortholog), under basal and insulin stimulating conditions were performed. We chose a variety of different cancer cell lines with active PI3K signaling pathways derived from different tissue sources. In total, we performed sixteen p85α endogenous antibody immunoprecipitation experiments with the non-small cell lung cancer (NSCL) lines EBC-1, HCC8227, A549, H1993 and H1703 and multiple myeloma cell lines H929 and RPMI-8226. (p110 was not immunoprecipitated as the antibodies do not precipitate well) from serum starved, serum fed and tyrosine kinase inhibitor drug treatments (PHA-665752 (c-Met inhibitor), Imatinib (ABL, PDGFR inhibitor), and Gefitinib (EGFR inhibitor)). These conditions and drugs are known to either activate or disrupt the PI3K complex and downstream AKT signaling. The human IPs were separated via a SDS-PAGE short gel run^11^ followed by in-gel digestion, and the TAP tagged *drosophila* IPs were digested in-solution and samples were cleaned using C_18_ ziptips. Gels were used to separate endogenous antibody contamination from the protein complex for the human cell IPs and this was not necessary for the drosophila TAP-tagged protein complex elutions since they are free from antibody contamination^32^,^35^. The peptide complex mixtures were analyzed by shotgun high resolution LC-MS/MS (Fig. 1A). Label-free quantification via spectral counting was used for relative protein abundance measurements. At least three spectral counts (two unique peptides) were required for protein identifications across the respective species datasets. Relative quantification was measured but not critical to the reciprocal BLAST overlap analysis. Therefore, a strategy was taken to uncover other potential interactions of biological significance. The drosophila Pi3k21b and Pi3k92e IPs using a tap-tagged system with a total of 202 (Supporting Data 1) unique drosophila proteins and the human IPs generated 522 (Supporting Data 2) total candidate proteins after common contaminants such as keratins, dermicidins, milk caseins, albumin, etc. were removed. Control LC-MS/MS experiments in drosophila from empty TAP vectors (Supporting Data 3) and human from rabbit IgG IPs (Supporting Data 4) were used to eliminate non-specific binding proteins such as ribosomal proteins, heat-shock proteins, actins, etc. and were subtracted from the subsequent reciprocal BLAST analysis since they are not part of the core PI3K complex. Next, database search results were combined to identify orthologous matches between fly and human using reciprocal BLAST. For gene products without orthologs, we used NCBI-Blastp to identify the reciprocal best three hits (with E-value < 1e-60 as the cutoff) between human proteomes and drosophila proteomes as the potential ortholog pairs. Note that the computational best fit for Pi3k21B is the splicing variant p55 gamma (p85 without SH3 and RhoGAP domain) and p85 alpha is the second best hit. A reciprocal BLAST analysis using the FlyBase dmel protein database and the EMBL-EBI IPI human database yielded 49 surviving proteins that are conserved across both human and *drosophila* and could have biological relevance (Fig. 1B). The 49 cross-species PI3K binding proteins (Table 1) included several key canonical associations with proteins such as the insulin receptor substrate 1 (IRS1; fly: chico; identified from PI3K IPs, not as a bait protein), platelet-derived growth factor receptor (PDGFR; fly: Pvr), insulin receptor (InR) and protein 14-3-3 (Fig. 1B). The full list of reciprocal blast hits with sample information, spectral count data and ratio information for each species can be found in Supplemental Data 5. Interestingly, other known mammalian PI3K adaptor proteins were missing from the BLAST overlap, such as the adaptor protein GAB2 (aka Daughter of Sevenless (Dos)), likely because the two or more pYXXM binding motifs required for p85 binding^36^ are not present in *Drosophila* DOS^37^.

**Figure 2:**
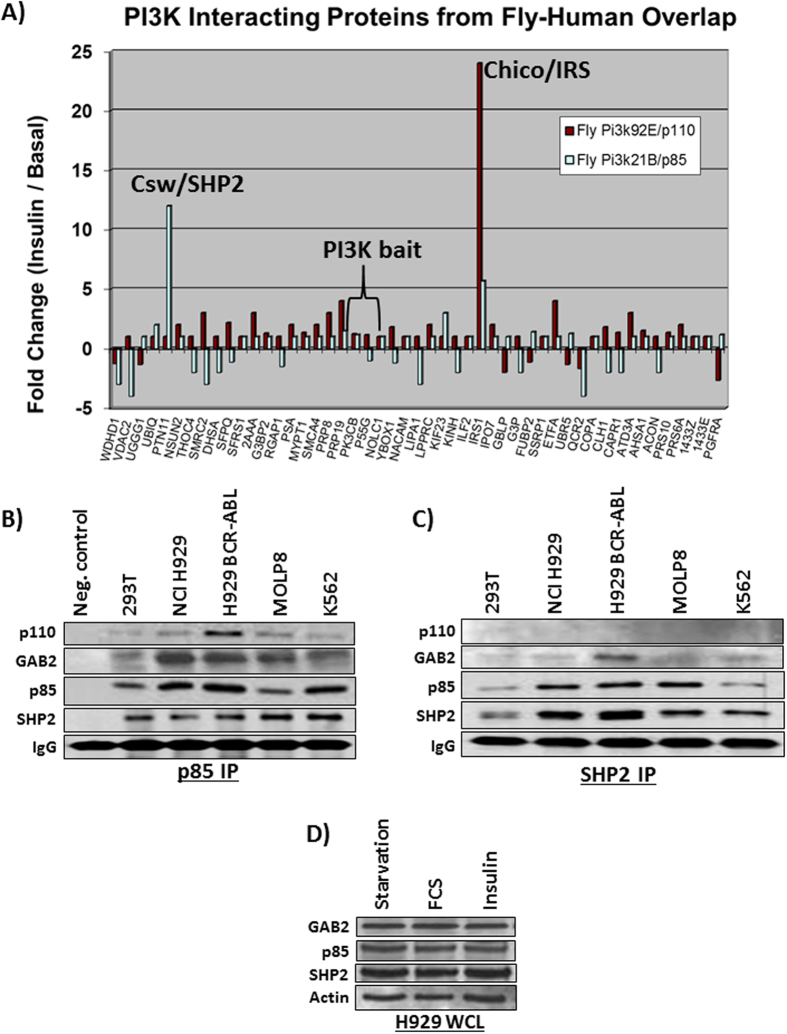
(**A**) The fold change of PI3K subunit IPs (Pi3k21b (p85) regulatory and Pi3k92e (p110) catalytic) in drosophila S2R+ cells. These 49 proteins represent the results of the human-drosophila reciprocal BLAST overlap analysis. Chico (IRS) is mainly associated with the catalytic subunit and Csw (SHP2) is mainly associated with the free regulatory subunit. **(B**) The p85 immunoprecipitation in 293T, NCI H929, BCR-ABL H929, MOLP8, and K562 cells and a mock IP control with no cell lysate immunoblotted for p110, GAB2, p85, SHP2 and IgG. **(C)** SHP2 immunoprecipitation in 293T, NCI H929, BCR-ABL H929, MOLP8, and K562 cells were immunoblotted for p110, GAB2, p85, SHP2 and IgG. All cell lines show a significant interaction of p85 with SHP2 even in the absence of p110. **(D)** Blots for p85, SHP2, GAB2 and actin control in H929 whole cell lysate under serum starved, full serum and insulin stimulation to test for protein expression levels of the core p85-SHP2-GAB2 ternary complex components.

In order to better understand the hits of interest, we focused on PI3K binding proteins with significant increase in binding affinity to PI3K upon insulin stimulation since it is directly involved in AKT activation. Fig. 2A showed the fold change of the 49 PI3K interacting proteins of the p85 and p110 IPs from fly cells treated with insulin and basal stimulation of which only Corkscrew (Csw/SHP2) and Chico (IRS) showed a significant increase in association with PI3K. While Chico was mainly detected with the Pi3k92e (p110) catalytic subunit, indicating a well-known interaction with the active PI3K heterodimer^38^, Csw (SHP2) mainly associated with Pi3k21B (p85), suggesting a high association with free p85, likely independent of AKT activation.

### Evaluation of the p85-SHP2 complex

The goal of further study was to investigate the binding and role of p85 with SHP2 in human cells since the importance of this interaction was significant and evolutionarily conserved (Fig. 2A). H929 cells have high ERK signaling but relatively low AKT signaling^39^. Previously published results showed that GAB2 is a major part of the PI3K complex in H929 cells^39^. While IRS2 can be detected at very low levels via p85 IP (less than three spectral counts) in H929, it is known that IRS proteins are only involved in the active p85/p110 PI3K heterodimer for AKT activation (Fig. 2A), therefore, we focused on the AKT independent complex that involved p85-SHP2-GAB2 to test the role of p85 associated SHP2 in the AKT and ERK pathways. We evaluated the p85 and SHP2 associated complex in BCR-ABL positive multiple myeloma H929 cells, NCI H929 cells, human embryonic kidney 293T cells, multiple myeloma MOLP8 cells and chronic myeloid leukemia K562 cells with immunoblots of p85, p110, SHP2 and GAB2 from p85 and SHP2 IPs (Fig. 2B,C) and the higher association of SHP2 with the p85 regulatory subunit of PI3K could be verified in all the cell lines. Taken together, these data established a core ternary complex of p85-SHP2-GAB2. Immunoblots for p85, SHP2 and GAB2 including the loading control actin in H929 whole cell lysate to prove that protein expression of the ternary complex was not altered under different conditions of serum starvation, fetal calf serum addition or insulin stimulation (Fig. 2D).

**Figure 3:**
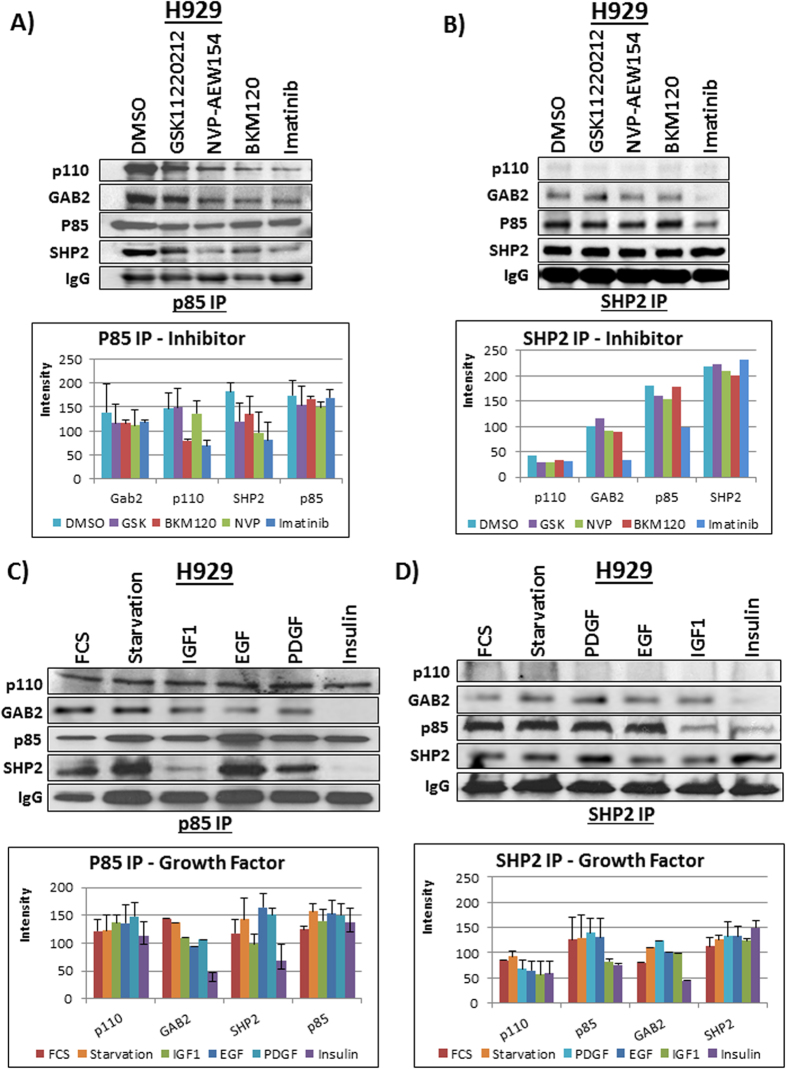
(**A**) The p85 IP in H929 cells was immunoblotted for p110, GAB2, p85 and SHP2 under treatment with various tyrosine kinase inhibitors (imatinib, BKM120, NVP-AEW154, and GSK11220212). The intensity of each band was calculated by measuring the band intensity with ImageJ and averaging the band intensity of duplicates. **(B)** The SHP2 IP in H929 cells with immunoblots for p110, GAB2, p85 and SHP2 under treatment with various kinase inhibitors (GSK11220212, NVP-AEW154, BKM120, imatinib).The blot showed the complex with GAB2 and p85 was only disrupted by imatinib and p110 was unable to bind the p85-SHP2 complex. **(C)** A P85 IP after stimulation with growth factors (FCS, Starvation, IGF1, EGF, PDGF, and Insulin) followed by immunoblots for p110, GAB2, p85 and SHP2 showed the disruption of the ternary complex only with insulin and IGF1. **(D)** A SHP2 IP after stimulation with growth factors (FCS, Starvation, PDGF, EGF, IGF1, and Insulin) followed by immunoblots for p110, p85, GAB2 and SHP2 showed the disruption of the ternary complex only with insulin and IGF1.

Figure 3A,B showed the p85 IP with immunoblots for SHP2, p110, GAB2 and p85 in BCR-ABL positive H929 cells after treatment with either DMSO vehicle or several kinase inhibitors (GSK11220212 (ERK inhibitor), NVP-AEW541 (IGF1R inhibitor), BKM120 (pan PI3K inhibitor), imatinib (ABL, PDGFR inhibitor)) to test the effect on the SHP2-p85 complex when either ERK or AKT signaling was inhibited. Only the treatment with imatinib disrupted the interaction of p85 and SHP2 in both ways, which down-regulates ERK1/2 phosphorylation in H929 cells^39^. Imatinib also disrupted the binding of SHP2 to GAB2 though the binding of p85 to GAB2 did not change significantly with imatinib treatment. Bar graphs were included in Fig. 3 to aid with interpretation. Additionally, the SHP2 IP in Fig. 3B showed a SHP2-p85-GAB2 complex formation independent of the p110 catalytic subunit of PI3K, a result suggesting that SHP2 was not involved in AKT activation^27^,^40^,^41^,^42^,^43^. Similar to the p85 IP, the SHP2 IP showed that the p85-SHP2 interaction was abrogated when BCR-ABL pathway was inhibited with imatinib, a result suggesting that ERK may be connected to the p85-SHP2 complex.

We also investigated the effects of the growth factors (activators) IGF1, PDGF, EGF, insulin and fetal calf serum (FCS) on the p85-SHP2-GAB2 complex (Fig. 3C,D). The p85 IP in Fig. 3C showed a strong interaction with SHP2 that was only disrupted by stimulation of the PI3K-AKT pathway via insulin or IGF1, which was also true for the reverse experiment of the SHP2 IP (Fig. 3D). The other activators did not have a significant effect on the p85-SHP2-GAB2 complex, suggesting that the ternary complex is not involved in AKT activation but still may play a role in ERK signaling. Interestingly, stimulation with insulin also disrupted the binding of GAB2 to p85 and GAB2 to SHP2, indicating that prior to insulin stimulation, p85, SHP2 and GAB2 may form a ternary complex. Due to the presence of BCR-ABL in H929 cells, the ERK pathway is constitutively activated^39^ with low AKT activation; however, AKT phosphorylation can be activated using IGF-1 or insulin treatment^44^, which coincided with a disruption of the p85-SHP2-GAB2 complex (Fig. 3C,D). This leads to the likelihood that the ternary complex of SHP2-p85-GAB2 may be involved in the ERK pathway^26^,^45^. Further, insulin might cause p85 and SHP2 to disrupt from the ternary complex, leading to the inability to activate the GRB2-SOS-Ras complex, thus abrogating ERK signaling.

It is important to mention that *drosophila* p85 (Pi3k21B) was bound to SHP2 (Csw) without GAB adaptors because *drosophila* GAB (Dos) does not contain multiple YXXM motifs, a previously accepted requirement for p85 interaction with GAB and other adaptor proteins^46^. This suggested that SHP2 can bind to p85 independent of GAB adaptors. SHP2 contains only a single YXXM motif in humans and no YXXM motif in *drosophila*, suggesting a direct binding mechanism for p85 binding to SHP2.

### SHP2 associated with free p85

**Figure 4:**
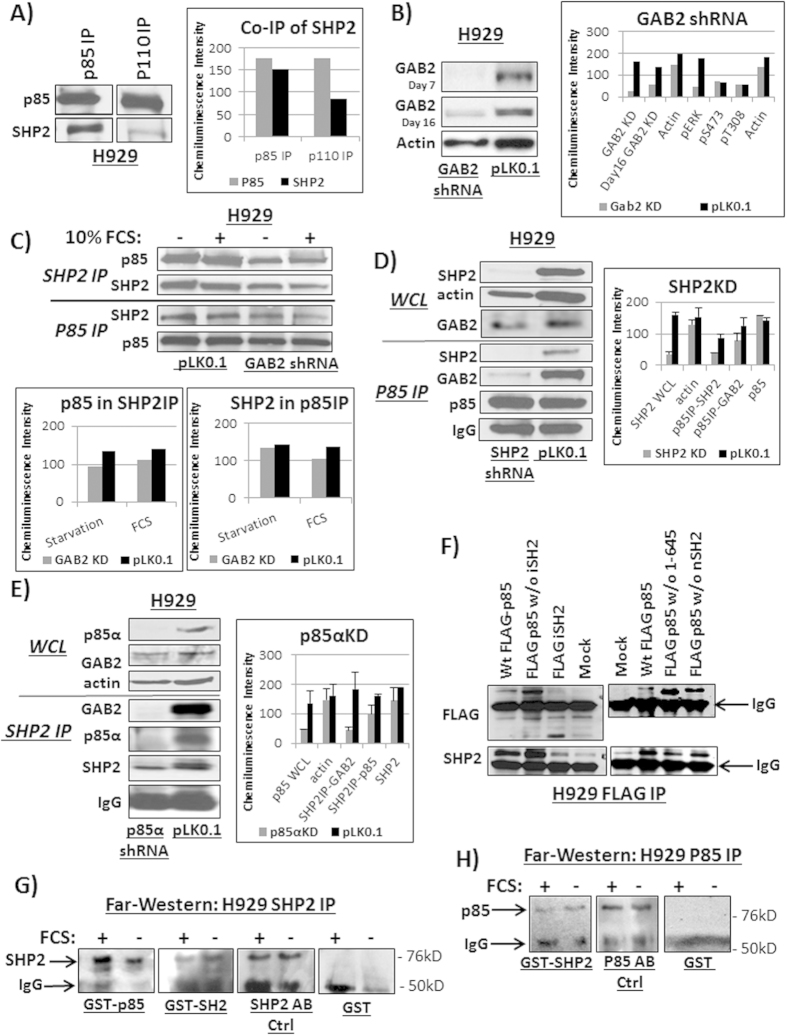
(**A**) The immunoblots of p85 (free p85 + p110 heterodimer) and p110 (only p110 heterodimer) IPs in H929 cells for quantification of SHP2 with equal p85 levels. The bar blots were generated by measuring the band intensity with ImageJ. **(B)** Immunoblots of GAB2 and actin control from stable GAB2 shRNA KD H929 cells. **(C)** IPs of p85 and SHP2 was performed in GAB2 KD and control cells the under starvation and serum (FCS) stimulation followed by blots for p85 and SHP2. **(D)** Immunoblots for SHP2, GAB2 and actin control from stable SHP2 shRNA KD of H929 cells. A p85 IP was performed in SHP2 KD cells and control H929 cells followed by blots for GAB2, SHP2, p85 and IgG control. **(E)** p85α, GAB2 and actin control immunoblots in stable p85α shRNA KD and control cells SHP2 IP was performed in p85 KD cells and control cells with blots for GAB2, SHP2, p85 and IgG control. **(F)** In H929 cells, FLAG tagged -p85 wt, FLAG tagged p85 without iSH2, FLAG tagged iSH2, FLAG tagged p85 without cSH2, FLAG tagged p85 without nSH2 and a negative control (mock) were transient transfected, followed by a FLAG IP. The eluent of the IP was blotted for FLAG peptide and SHP2. **(G)** Far-Western blots were performed from SHP2 IPs of serum stimulated or starved cells incubated with either GST-tagged p85, GST- tagged SH2 domain, SHP2 antibody or GST control followed by secondary antibody. The tagged p85 and the GST- tagged SH2 domain but not the control binds positively to SHP2 under both conditions. **(H)** Far-Western blots were performed by from p85 IPs of serum stimulated or starved cells incubated with a GST-tagged SHP2, p85 antibody or GST control followed by secondary antibody against the GST tag. The tagged SHP2 but not the control binds positively to p85 under both conditions.

The SHP2 IPs in Fig. 3 suggested that the p110 PI3K catalytic subunit (required for AKT activation) was not involved in the p85-SHP2 complex, thus the complex is AKT independent. In order to verify this, the p85 and p110 PI3K subunits were immunopurified separately and immunoblotted various aliquots in order to obtain equal amounts of p85. The p85 IP represented a mix of both free p85 (inactive PI3K) and p85 bound to p110 (PI3K heterodimer that is required for AKT activation) while the p110 IP represented p85 completely bound to p110. The immunoblot of both IPs showed a higher level of SHP2 associated with p85 from the p85 IP (mixture of active and inactive PI3K) than from the p110 IP (active PI3K) (Fig. 4A), showing that SHP2 complexed with inactive and free p85.

### shRNA knock down demonstrated the direct interaction of p85 and SHP2 independent of GAB2

Our results indicated the ternary complex of SHP2, p85 and GAB2 formed independently of p110, however, the role of GAB2 was unclear. We tested whether a stable knockdown of each member affected the ternary complex in H929 cells. First, a stable knockdown of GAB2 with a short hairpin RNA (shRNA) pool resulting in ~80% efficiency, which is visible in the whole cell lysate with and without knock down immunoblotted for both GAB2 and actin as a loading control (Fig. 4B). The interaction of the remaining members of the complex was hardly affected by GAB2 knock down (Fig. 4C), as IPs for p85 or SHP2 under starved and normal growth conditions did not reveal a loss in SHP2-p85 interaction. Next, we stably knocked down SHP2 with 90% efficiency, which is evident in the whole cell lysate with and without knock down immunoblotted for SHP2, GAB2 and actin (Fig. 4D). The p85 IP from SHP2 KD showed an abrogated association with GAB2, indicating that SHP2 possibly mediated the interaction of p85 with GAB2 in H929 cells. We attempted to study the double knock down of p85α and p85β in H929 cells but cells did not survive longer than 7 days. Thus, we decided to focus on the knockdown of p85α subunit only in H929 whole cell lysate (90% efficiency) and immunoblotted for p85α, GAB2 and actin as a loading control (Fig. 4E). The SHP2 IP could not pull down GAB2 in the absence p85α, suggesting that p85 was essential for the p85-GAB2-SHP2 ternary complex (Fig. 4E). These results were supported by the *drosophila* LC-MS/MS data where p85 was able to bind SHP2 directly without GAB adaptors. These knockdown data concluded that the p85-SHP2 interaction occurs prior to the formation of the ternary complex with GAB2 since GAB2 was unable to interact with p85 or SHP2 alone.

### Transfected FLAG-p85 showed SHP2 interacted with the SH2 domains on p85

We next investigated which domain of p85 was involved in the direct interaction with SHP2. FLAG-tagged constructs (wt-p85, p85 without iSH2, iSH2 only, p85 without cSH2 and p85 without nSH2) of p85 were generated and transiently transfected into H929 cells. Three days after transfection, the cells were lysed and immunoprecipitated using an antibody against the FLAG-tag and immunoblotted for FLAG and SHP2 (Fig. 4F). The constructs of wild type p85 as well of p85 lacking inter-SH2 domain could equally IP SHP2, which suggested that the iSH2 domain was not involved in SHP2-p85 binding. This was verified with the FLAG tagged iSH2 domain-only construct, which was not able to IP SHP2 (Fig. 4F). Both constructs containing only one SH2 domain (p85 without cSH2 and p85 without nSH2) co-immunoprecipitated SHP2, although compared the wild type p85, the intensity of the SH2 band was lower because only half of the SH2 domains in the p85 construct were available for SHP2 binding. These data demonstrate the importance of the p85 SH2 domain in the interaction with SHP2.

### Bacterially expressed proteins showed direct interaction between SHP2 and p85 independent of adaptors

We further investigated whether the interaction of p85 with SHP2 was independent of any other unidentified adaptors and whether we could verify the SH2 domain as the binding domain for SHP2. Therefore, we analyzed the direct interaction of p85 and SHP2 using the Far-Western blot technique whereby proteins are bound directly to proteins that were run in gel and transferred to a membrane^47^. We first immunopurified SHP2 from H929 cells in full serum or serum starved conditions and incubated the blot with either full length (FL) glutathione-s-transferase (GST)-p85, GST-SH2 construct or GST-control followed by a primary antibody against the GST peptide and a secondary antibody (Fig. 4G). The GST-p85 marked a strong band at the same position as SHP2 confirming the direct interaction between both proteins without any additional adaptors. Furthermore, GST-SH2 showed a band at SHP2, though with a weaker signal but still verifying the ability of SHP2 to bind this domain. We also immunopurified p85 from H929 cells in full serum or serum starved conditions and the blot was incubated with FL GST-SHP2, followed by a primary antibody against the GST peptide and a secondary antibody (Fig. 4H). Again, the interaction of transferred endogenous p85 with GST-tagged SHP2 was able to bind directly under both conditions. The incubation with GST control alone did not show any interaction (Fig. 4G,H). These data showed that the interaction of SHP2 and p85 was not mediated by adaptors and that the SH2 domain on p85 was mainly responsible for the SHP2 binding.

### Model for p85-SHP2 complex in H929 cells

**Figure 5:**
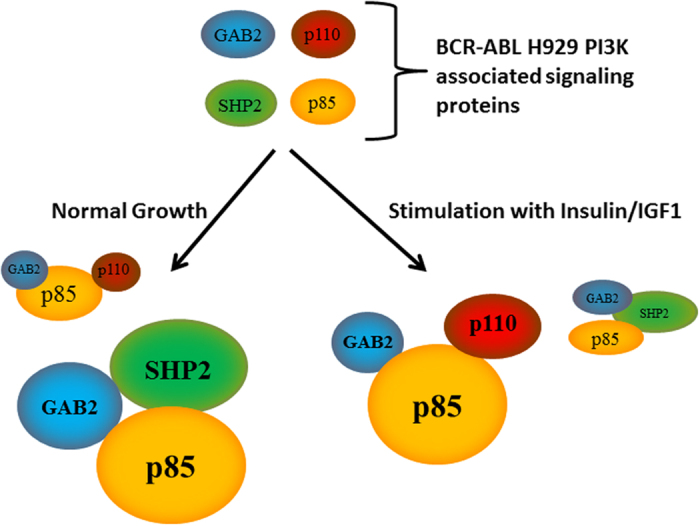
Model for BCR-ABL positive H929 signaling and the SHP2-p85 direct complex. Under normal growth conditions, SHP2 is bound to the majority of p85 on its SH2 domains initiating a complex with GAB2. A minor portion of p85 forms the PI3-kinase with p110 and GAB2 to produce low levels of AKT activity. When H929 cells is stimulated with insulin, p85 dissociates from SHP2 resulting in a disruption of the SHP2-p85-GAB2 complex. This causes p85 to mainly form a heterodimer complex with p110, resulting in AKT activation.

We proposed that under normal growth conditions without stimulation, p85 binds directly to SHP2 over the SH2 domains to ultimately form a ternary complex with GAB2. This is the AKT independent complex which can be associated with ERK signaling. In addition, a subpopulation of p85 could be found in a complex with p110 and GAB2, a complex involved in AKT signaling (Fig. 5). Stimulation with insulin or IGF1 disrupted the GAB2-SHP2-p85 ternary complex. This process released p85, allowing it to bind to p110 to form active PI3K.

## Discussion

Since many important protein-protein interactions are critical for cellular regulatory functions are conserved through evolution, we performed a cross-species proteomics comparison of PI3K interacting proteins. Using this strategy, we could assess the core biology that was conserved through evolution and looked past non-specific binding proteins that are common to proteomics PPI experiments. By analyzing and comparing protein complexes by shotgun LC-MS/MS in *drosophila* and mammalian cancer cells, the unusually strong binding of SHP2 (Csw) to p85 (Pi3k21B) was identified. The biology of PI3K showed that SHP2 played a distinct role in a ternary complex with the GAB2 adaptor and the p85 regulatory component of PI3K in BCR-ABL positive H929 multiple myeloma cancer cells. Only stimulation with insulin after starvation disrupted the ternary complex of p85-SHP2-GAB2, which led to stimulation of free p85 regulatory subunit to form a complex with p110 catalytic subunit of PI3K for downstream AKT activation^48^,^49^,^50^,^51^. Additionally, while most kinase inhibitors had little effect on the p85-SHP2-GAB2 complex, the treatment with imatinib partially disrupted the complex. This is likely due to inhibition of the BCR-ABL driving kinase and the downstream ERK pathway, causing the release of p85 from SHP2 and GAB2 and its association with the BCR-ABL complex. Furthermore, only p85, but not p110, was detectable in an SHP2-IP by both LC-MS/MS and immunoblots. These data suggest a role for SHP2 as a free cytosolic p85 adaptor that was released when the PI3K pathway was activated^26^. Moreover the knockdown of GAB2 and the Far-Western results imply the interaction of SHP2 with p85 was formed directly on the SH2 domains of p85 without the need of GAB2 or any other adaptor proteins. It is not known whether SHP2 directly regulates AKT via PI3K signaling, though SHP2 is known to be critical to MAPK/ERK signaling^52^. The direct interaction of SHP2 with p85 had generally been overlooked as the SH2 domains of both p85 (PI3K) and SHP2 bind directly to phosphotyrosine residues on GAB and IRS proteins^53^,^54^,^55^.

The data also imply that free p85 may be involved in the ERK pathway via direct binding to SHP2 via the p85 SH2 domains that initiate a complex with GAB2 as an adaptor. While this was not the first cross-species omics investigation to be presented, it did address for the first time, the direct interaction of a core signal transduction complex (p85-SHP2), widely implicated in cancers. These data showed the power of using model species together with mammalian species coupled with high resolution mass spectrometry for uncovering conserved biological function.

## Additional Information

**How to cite this article**: Breitkopf, S. B. *et al.* A Cross-Species Study of PI3K Protein-Protein Interactions Reveals the Direct Interaction of P85 and SHP2. *Sci. Rep.* **6**, 20471; doi: 10.1038/srep20471 (2016).

## Supplementary Material

Supplementary Dataset 1

Supplementary Dataset 2

Supplementary Dataset 3

Supplementary Dataset 4

Supplementary Dataset 5

## Figures and Tables

**Table 1:** List of Common PI3K Associated Proteins from *drosophila*-Human Reciprocal Blast.

*Drosophila* Accession Number	*Drosophila* Molecular Weight	Protein Name (Human)	Human Accession Number	Human Molecular Weight
FBpp0086732	97 kDa	WD repeat and HMG-box DNA-binding protein 1	WDHD1	126 kDa
FBpp0079771	31 kDa	Voltage-dependent anion-selective channel protein 2	VDAC2	32 kDa
FBpp0074831	174 kDa	UDP-glucose:glycoprotein glucosyltransferase 1	UGGG1	177 kDa
FBpp0079606	18 kDa	Ubiquitin	UBIQ	9 kDa
**FBpp0070362**	**92 kDa**	**Tyrosine-protein phosphatase non-receptor type 11**	**PTN11/SHP2**	**68** kDa
FBpp0070637	84 kDa	tRNA (cytosine-5-)-methyltransferase NSUN2	NSUN2	86 kDa
FBpp0081156	28 kDa	THO complex subunit 4	THOC4	27 kDa
FBpp0082692	131 kDa	SWI/SNF complex subunit SMARCC2	SMRC2	133 kDa
FBpp0085736	72 kDa	Succinate dehydrogenase [ubiquinone] flavoprotein subunit, mitochondrial	DHSA	73 kDa
FBpp0074011	77 kDa	Splicing factor, proline- and glutamine-rich	SFPQ	76 kDa
FBpp0082724	28 kDa	Splicing factor, arginine/serine-rich 1	SFRS1	28 kDa
FBpp0099974	65 kDa	Serine/threonine-protein phosphatase 2A 65 kDa regulatory subunit A alpha isoform	2AAA	65 kDa
FBpp0082264 (+4)	75 kDa	Ras GTPase-activating protein-binding protein 2	G3BP2	54 kDa
FBpp0086767	70 kDa	Rac GTPase-activating protein 1	RGAP1	71 kDa
FBpp0072581 (+5)	99 kDa	Puromycin-sensitive aminopeptidase	PSA	103 kDa
FBpp0075196	124 kDa	Protein phosphatase 1 regulatory subunit 12A	MYPT1	112 kDa
FBpp0075278	185 kDa	Probable global transcription activator SNF2L4	SMCA4	185 kDa
FBpp0087124	280 kDa	Pre-mRNA-processing-splicing factor 8	PRP8	274 kDa
FBpp0085902	55 kDa	Pre-mRNA-processing factor 19	PRP19	55 kDa
FBpp0083348	127 kDa	Phosphatidylinositol-4,5-bisphosphate 3-kinase catalytic subunit beta isoform	PK3CB	123 kDa
**FBpp0077791**	**57** kDa	**Phosphatidylinositol 3-kinase regulatory subunit gamma (BAIT)**	**P55G/P85A**	**54** kDa**/84** kDa
FBpp0088542	71 kDa	Nucleolar phosphoprotein p130	NOLC1	74 kDa
FBpp0075759	38 kDa	Nuclease-sensitive element-binding protein 1	YBOX1	36 kDa
FBpp0086971	23 kDa	Nascent polypeptide-associated complex subunit alpha, muscle-specific form	NACAM	221 kDa
FBpp0078930	135 kDa	Liprin-alpha-1	LIPA1	136 kDa
FBpp0080637	157 kDa	Leucine-rich PPR motif-containing protein, mitochondrial	LPPRC	158 kDa
FBpp0073083	101 kDa	Kinesin-like protein KIF23	KIF23	110 kDa
FBpp0086328	110 kDa	Kinesin-1 heavy chain	KINH	110 kDa
FBpp0082066	44 kDa	Interleukin enhancer-binding factor 2	ILF2	43 kDa
FBpp0079677	108 kDa	Insulin receptor substrate 1	IRS1	132 kDa
FBpp0076408	119 kDa	Importin-7	IPO7	120 kDa
FBpp0079187	36 kDa	Guanine nucleotide-binding protein subunit beta-2-like 1	GBLP	35 kDa
FBpp0087977	35 kDa	Glyceraldehyde-3-phosphate dehydrogenase	G3P	36 kDa
FBpp0086220	82 kDa	Far upstream element-binding protein 2	FUBP2	73 kDa
FBpp0072151	82 kDa	FACT complex subunit SSRP1	SSRP1	81 kDa
FBpp0087186	34 kDa	Electron transfer flavoprotein subunit alpha, mitochondrial	ETFA	35 kDa
FBpp0081568	319 kDa	E3 ubiquitin-protein ligase UBR5	UBR5	309 kDa
FBpp0075069	45 kDa	Cytochrome b-c1 complex subunit 2, mitochondrial	QCR2	48 kDa
FBpp0072693	139 kDa	Coatomer subunit alpha	COPA	138 kDa
FBpp0073966 (+5)	191 kDa	Clathrin heavy chain 1	CLH1	192 kDa
FBpp0074865	104 kDa	Caprin-1	CAPR1	78 kDa
FBpp0082728	68 kDa	ATPase family AAA domain-containing protein 3A	ATD3A	71 kDa
FBpp0085258	40 kDa	Activator of 90 kDa heat shock protein ATPase homolog 1	AHSA1	38 kDa
FBpp0081002	85 kDa	Aconitate hydratase, mitochondrial	ACON	85 kDa
FBpp0070890	44 kDa	26S protease regulatory subunit S10B	PRS10	44 kDa
FBpp0083843	48 kDa	26S protease regulatory subunit 6A	PRS6A	49 kDa
FBpp0087500 (+4)	28 kDa	14-3-3 protein zeta/delta	1433Z	28 kDa
FBpp0082990	30 kDa	14-3-3 protein epsilon	1433E	29 kDa
FBpp0079244 (+1)	170 kDa	Platelet-derived growth factor receptor alpha	PGFRA	123 kDa
